# Bibliometric Analysis on Geriatric Nursing Research in Web of Science (1900–2020)

**DOI:** 10.1155/2021/8758161

**Published:** 2021-09-28

**Authors:** Arezoo Ghamgosar, Maryam Zarghani, Leila Nemati-Anaraki

**Affiliations:** ^1^Department of Medical Library and Information Sciences, School of Health Management and Information Sciences, Iran University of Medical Sciences, Tehran 1996713883, Iran; ^2^Medical Biotechnology Research Center, School of Paramedicine, Guilan University of Medical Sciences, Rasht 44817, Iran; ^3^Department of Medical Library and Information Sciences, School of Health Management and Information Sciences, Iran University of Medical Sciences, Tehran, Iran

## Abstract

**Objective:**

Aging is a growing public health concern for people, organizations, and governments. The current study was undertaken to provide insights into the global research output on geriatric nursing.

**Methods:**

A bibliometric study was implemented using the WoS database for the period from 1900 to 2020. Various tools and measures were used to analyze and visualized.

**Results:**

The search strategy found 4923 papers. The oldest paper was written by Beverly C. Andre in 1953. As team size increases, so does the number of citations. The USA was the active country and the highest number of coauthors. New York University was an active institution. Stig Karlsson was the most active author in Geriatric Nursing with 28 articles from Sweden, followed by Koen Milisen and Sandman, with 26 articles each from Sweden and Belgium. The most frequent words in this field were depression, malnutrition, education, Alzheimer's disease, and dementia. The latest research themes in this field were COVID-19, interprofessional locomotive syndrome, emergency nursing, and public health. The most influential papers were specified. Journal of the American Geriatrics Society was the most active journal.

**Conclusions:**

Geriatric nursing is a rooted field and has received special attention in the last decade. Policymakers, especially in developing countries, should pay attention to geriatric nursing as a specialty of nursing to solve aging issues they would face considering the increasing elderly population.

## 1. Introduction

Aging is the most complex human phenotype and a health-related concern for individuals, organizations, and governments [1]. It is essentially a biological phenomenon and is now a global issue [2, 3]. Aging, which is widely defined as a reduction in the time-dependent function of organs, has affected most living things and has created curiosity and excitement throughout human history [4]. The United Nation has a global estimate of 703 million individuals aged >65 years based on statistics provided by the World Population Prospects 2019 (United Nations, 2019), indicating an increase from 6% in 1990 to 9% in 2019. The population of the elderly is estimated to reach 1.5 billion in 2050 (16%, one in six people) [5]. Therefore, governments are rapidly implementing policies that suit the phenomenon of aging in the population [6].

In response to these facts as a strategy, the health systems of different countries have paid special attention to the role of geriatric nursing [7]. Geriatric nursing emerged as a specialty after the 1950s. In meeting the needs of the elderly, the need for trained nurses with specialized scientific training to meet the care needs of the elderly was essential [8, 9]. This interest was followed by a great desire to conduct scientific research in this field [10]. Therefore, during the past years, scientific production in the field of geriatric nursing has increased [4, 11] and has now flourished [12].

Bibliometrics has become a central component of research evaluation [13]. Bibliometric analysis can offer a general, quantitative, and qualitative overview of a specific topic with a vast background. In particular, it can express the evolution and trend of scientific development in a field, identify the advantages and newly emerging ideas, and evaluate the achievements and influence of countries, institutions, journals, papers, and individuals [14]. Bibliometrics as a global standard is the crossdisciplinary science of quantitative analysis of all knowledge carriers by mathematical, bibliographical, and statistical methods [15, 16]. It plays a significant role, as a valuable research method, for quantifying scientific works, discovering interactions among scientists, and understanding recent advances in research [17]. Its methodologies generally use information technology to process and analyze quantitative and qualitative data from bibliographic information and provide meaningful implications [18]. The number of citations is the most frequently used indicator in evaluating the quality of papers, researchers, research centers, and universities [19] and evaluating the impact of an article on the existing literature [20].

Accordingly, the Web of Science (WoS) is often used for performing bibliometric analyses. The present study is the first comprehensive scientometric study of geriatric nursing. Evaluation of texts and presenting a comprehensive image of texts with respect to issues such as description, trend, and citation analysis of results, the value of team size, active countries, active institutions, international research collaboration, prolific authors, research networks of authors, research themes, most cited papers, and active journals can expand and produce new knowledge.

The findings are valuable for geriatric nursing researchers also for policy decision makers, top-level managers, politicians, and academic librarians. To the best of our knowledge, there is no bibliometric study and visualization map on geriatric nursing literature. Herein, this study fills this gap in the literature by conducting a bibliometric study of the geriatric nursing analysis.

## 2. Materials and Methodology

### 2.1. Data Source

A bibliometric study was implemented using the Web of Science (WoS) Core Collection for the period from 1900 to 2020. The WoS core collection database is a selective citation index of scientific and scholarly publishing consisting of journals, proceedings, books, and data compilations. The WoS is a selective, structured, and balanced database, not merely a catalog of publications. It has complete citation linkages and enhanced metadata supporting various information needs. Data are used by the WoS for mainly research performance evaluation, including rankings, mapping topics and monitoring trends, and investigating aspects of the history and sociology of science and scholarly activity [21].

### 2.2. Search Strategy

The WoS database was searched in 7 November 2020. Medical subject headings were used and followed recommendations of terms for search. In order to cover all the appropriate search terms, systematic review studies of previous geriatric nursing were used, and geriatric nurses were consulted to obtain additional terms used in practice [2, 10]. The search terms that would be used to create a search query were then finalized. The search strategy was designed with search terms including (senior∗ OR aged∗ OR Elder∗ OR Geri∗OR age∗ OR “OlderAdult∗” OR geriatric∗ OR aging OR “very elderly” OR “frail elderly” OR “aged 80 and over”) AND (geriatric nurs∗ OR gerontological nurs∗ OR gerontologic nurs∗), while being limited to the field of “Topic.” The field of “language” was limited to “English,” and the field of “document types” was limited to “article.” Only original research articles were included, and other types of articles such as letters to editors, reports, notes, short communications, short surveys, patents, and undefined were excluded.

### 2.3. Data Collection

The authors used the following indicators: description, trend, and citation analysis of results, the value of team size, active countries, active institutions, international research collaboration, prolific authors, research networks of authors, research themes, most cited papers, and active journals. After data collection, all data were exported in plain text and Excel format. The record content was limited to the full record and cited references. After this stage, WoS options, Microsoft Excel, version 2013, and some bibliometrics software, including Bibexcel, VOSviewer program (version 1.6.7) (Leiden University, Leiden, The Netherlands), were used.

### 2.4. Bibliometric and Visualized Analysis

To determine description, trend, and citation analysis of results, the online option of the WoS and Bibexcel, version 2008-08 was used. Using Bibexcel and by calculating the correlation between the number of authors and median citation to each article, the team size value was extracted. In order to receive active countries, active institutions, and prolific authors, we used bibliographic coupling analysis. A python script was written to unify author names with different spellings. The authors applied the VOSViewer software program (version 1.6.7). This software displays the area of international research collaboration, research networks of authors, and author keyword visualization of literature on research themes [22]. To obtain the most cited papers, after performing the search strategy, the retrieved results were limited to the field of “time cited-highest to lowest” and sorted accordingly. The authors applied the Bradford Law to obtain active journals in the field of geriatric nursing [23]. According to this bibliometric law, a small number of scientific journals is responsible for the great majority of publications on specific different themes and thus correspond to the core of scientific production [24]. Initially, 990 journals covering 4923 articles were divided into three categories so that the number of articles in all categories will be approximately equal. The first core was then introduced as the Bradford core.

## 3. Results

### 3.1. Description, Trend, and Citation Analysis of Results

**Figure 1:**
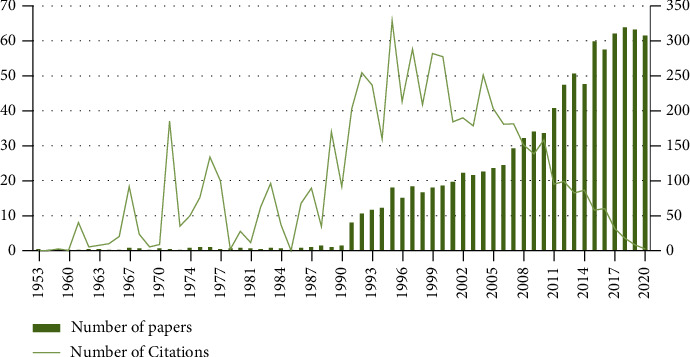
Number of papers and number of citations of Geriatric Nursing (1990–2020).

The search strategy found 4923 total papers and an average of 158 papers per year. The retrieved papers received 112,461 citations, an average of 22.91 citations per paper. The total citing papers were 77,196. The *h*-index of the retrieved papers was 125 (Figure 1). The oldest paper was written by Beverly C. Andre in 1953 entitled “Nursing Programs in Schools of Nursing” in the Journal of Gerontology [25]. His affiliation is Department of Nursing, Northwestern State College Natchitoches, Louisiana.

### 3.2. Value of Team Size

**Figure 2:**
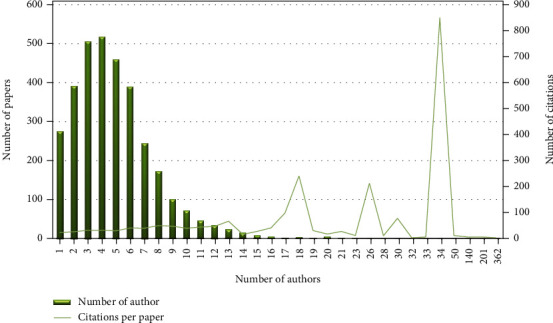
Team size and number of citations of Geriatric Nursing (1990–2020).

Figure 2 shows the team size distribution and the average citation of each document in geriatric nursing. 18215 authors have contributed to 4923 scientific papers in the field of geriatric nursing. Accordingly, the pattern of coauthorship in this field was four, which means that 15% of scientific articles in this field have been written in collaboration with a team of four people. By removing outdated data in this subject area, there was a correlation between team size and average citations of 0.29, which means that as team size increases, so does the number of citations. The highest average number of citations was related to an article by 34 authors of international cooperation, and 601 received citations. The title of this article is “Defeating Alzheimer's disease and other dementias: a priority for European science and society”, published in The Lancet Neurology in 2016. Studies on patient care and related research, from basic to clinical, in Alzheimer's disease and other dementias should be organized in the future [26].

### 3.3. Active Countries

Ninety-three countries contributed to geriatric nursing papers, with 1862 (37.822%) papers published in the USA, followed by Canada with 307 (6.236%) and Australia with 292 (5.931%). In terms of geographical distribution, three countries from the Americas, the countries that comprise the regions of North, Central, and South America, six countries from Europe, and one country from Oceania were the most active. These countries account for almost 81% of all articles (Table 1).

### 3.4. Active Institutions

Institutions affiliated with American universities were the most active in this field. The next rankings were held by Canadian and Swedish universities. The top ten active institutions contributed to 693 papers (12.755%). The top active institutions included eight in North America and two in Europe. New York University came in first with 97 articles, followed by the University of California Los Angeles with 93, the University of Toronto with 76, and the Karolinska Institutet with 65 (Table 2).

### 3.5. International Research Collaboration

**Figure 3:**
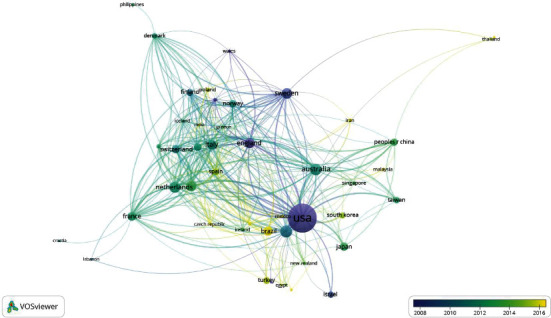
Visualization of international research collaboration with a minimum of 5 papers on Geriatric Nursing (1990–2020). For each of the 49 countries, the total strength of the coauthorship links with other countries will be calculated. The countries with the greatest total link strength will be selected.

Figure 3 shows a visualization map of international research collaboration among countries. The USA, Canada, and Australia are the countries that have published the largest number of scientific articles with 1786, 295, and 278 articles, respectively. Scientific papers from Northern Ireland, Scotland, Wales, the United Kingdom, and the USA have a higher average age and the countries with a background in this field. Indonesia, Mexico, Iran, Jordan, and Malaysia are also among the countries that have recently started publishing research articles in the field of geriatric nursing.

Figure 3 also reveals the coauthorship clustering of countries that have published more than five scientific articles in the field of geriatric nursing. Accordingly, forty-nine countries are located in nine clusters. Countries that have the same color are in the same cluster and have more allies. The USA is in purple with Japan, South Korea, and Taiwan, with the highest number of coauthors. Also, Canada is in another brown cluster with the highest number of coauthors with Israel and New Zealand. Most of Australia's counterparts are also dark blue, with Indonesia, Iran, Malaysia, China, Singapore, and Thailand, all of which are from Asia, especially Southeast Asia.

### 3.6. Prolific Authors

Stig Karlsson was the most active author in geriatric nursing with twenty-eight articles from Sweden, affiliated as professor at the department of nursing, followed by Koen Milisen and Per-Olof Sandman, with twenty-six articles each from Sweden, affiliated as professor at the department of nursing and Belgium, affiliated as professor at department of geriatric medicine (Table 3).

### 3.7. Research Networks of Authors

**Figure 4:**
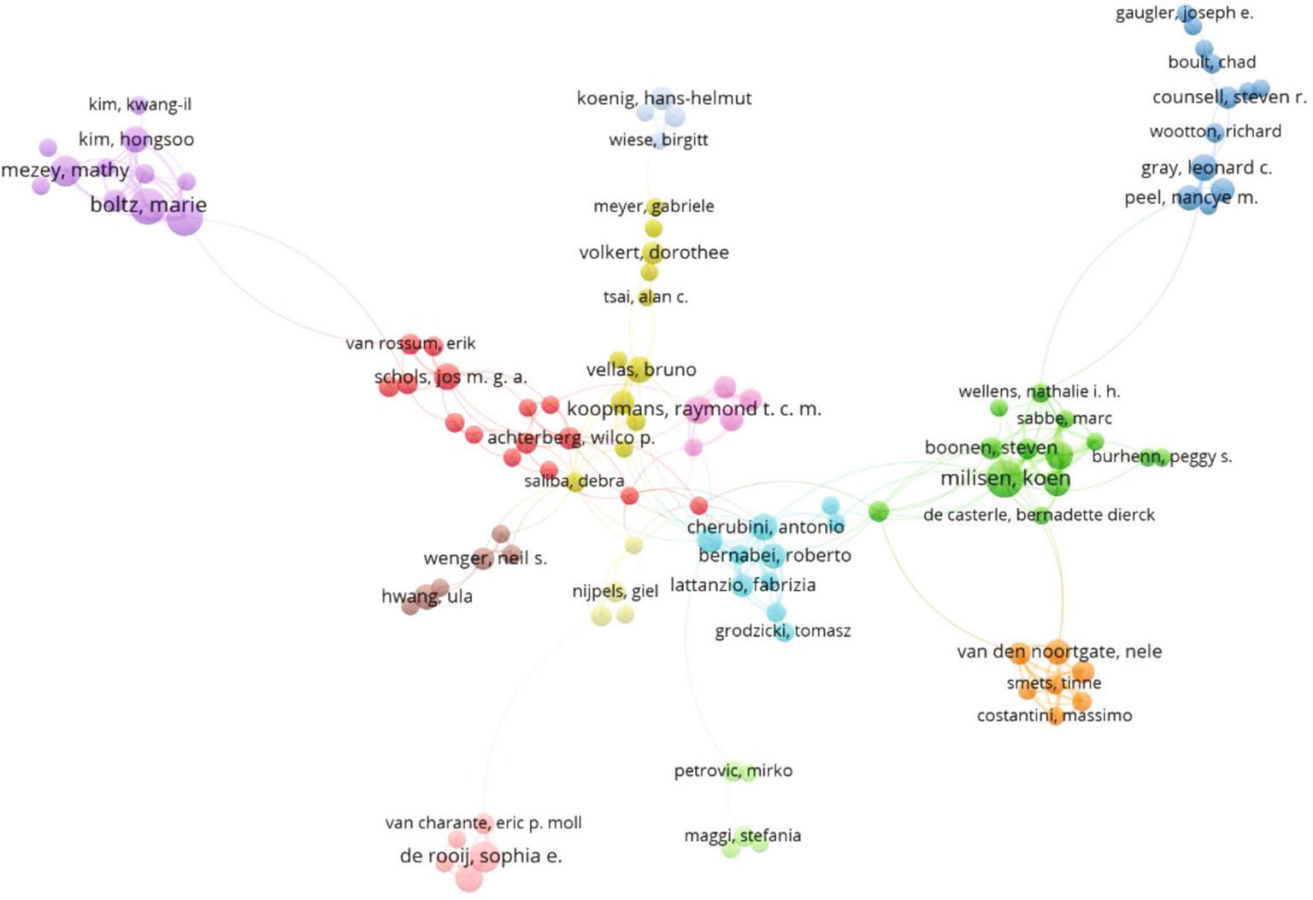
Visualization of research networks of authors with a minimum of 5 papers on Geriatric Nursing (1990–2020). Authors in the same cluster had close research interests.

Figure 4 shows the clustering of authors who have published more than five scientific articles. In this figure, the size of each circle indicates the number of scientific articles by the author. This network is composed of 1268 authors. The closer the items are, the more relevant they are. Accordingly, as shown, 173 authors are in sixteen clusters.

### 3.8. Research Themes

**Figure 5:**
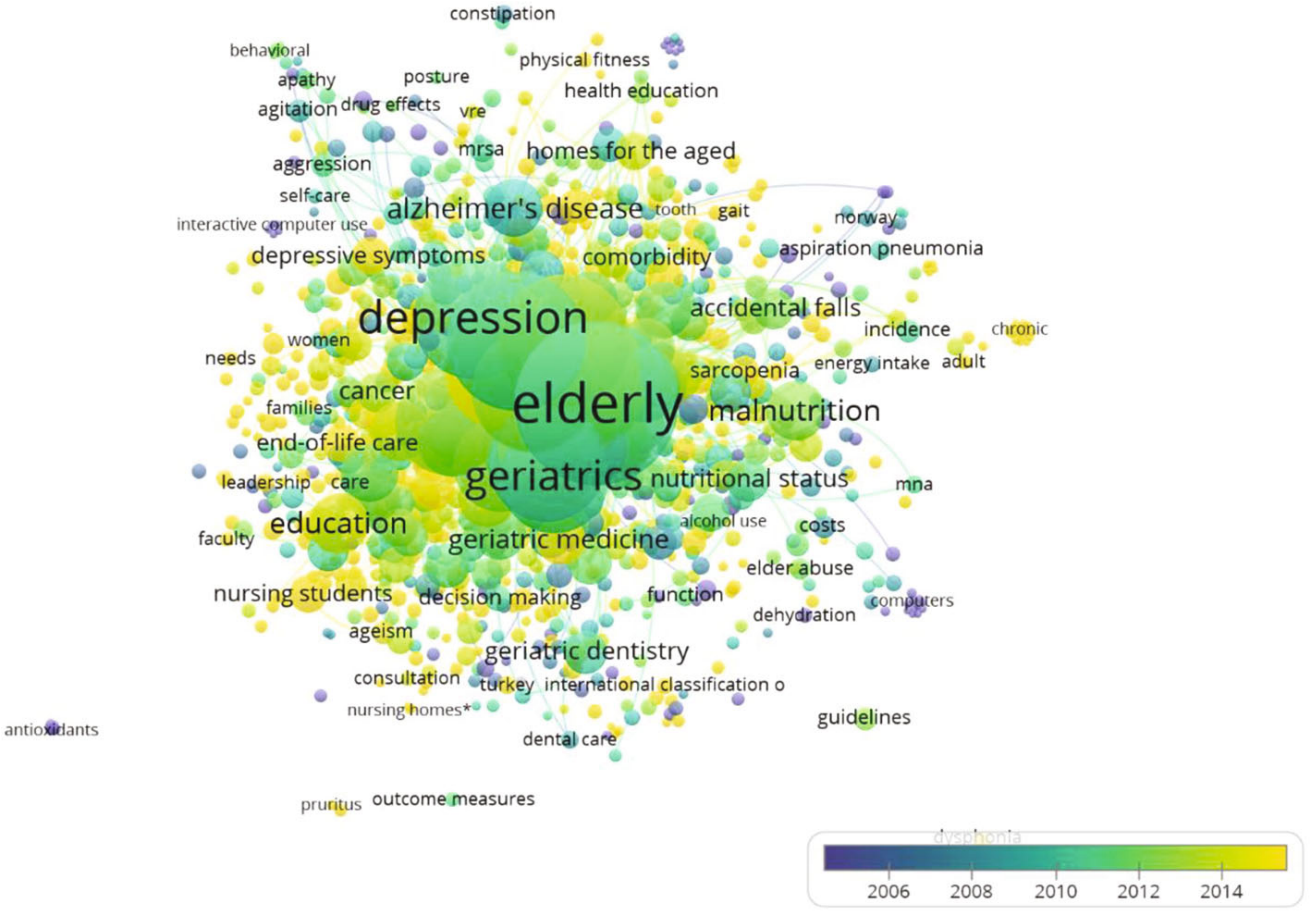
Network visualization map of most frequent terms in authors' keywords of the retrieved literature on Geriatric Nursing (1990–2020).

The content of the articles was analyzed by analyzing authors' keywords in order to extract the general themes of the studies.  Figure 5 shows the keywords of the authors in the field of geriatric nursing. Accordingly, by deleting the words “elderly” and “aged,” the most frequent words in this field were depression, malnutrition, education, Alzheimer's disease, and dementia, respectively. The closer the keywords get from dark blue to yellow, the newer the words are to each other. Therefore, the latest research themes in this field were COVID-19, interprofessional locomotive syndrome, emergency nursing, and public health.

### 3.9. Most Cited Papers

Table 4 shows the eight most cited papers were published in the 1990s, and only two articles in 2004 and 2009 were among the most cited papers.

### 3.10. Active Journals

Table 5 introduces the twenty journals with the highest frequency in the publication of Geriatric Nursing in the WoS database. The Journal of the American Geriatrics Society was most active, followed by the Journal of American Medical Directors Association and Geriatric Nursing with a significant difference. The share of twenty active journals was 1763 articles in total. Among these journals, fifteen were related to geriatrics, three to nursing, one to psychogeriatrics, and one to medical sciences.

## 4. Discussion

This paper presents the first bibliometric analysis study of trends in geriatric nursing scholarship. Over the period of sixty-seven years, 4,923 articles in the field of geriatric nursing were described and cited, and some concepts were visualized for better understanding. The chart of publication of articles (Figure 1) showed that nursing is a discipline with a long history. According to the WoS database, the term geriatric nursing was first used in 1953 in an article entitled “Geriatric Nursing Programs in Schools of Nursing” [25]. The *h*-index of the retrieved papers on geriatric nursing was 125, which is higher than MERS-CoV [27] and Elder Abuse [28]. This indicates that the articles published over a period of time have been of considerable interest to readers and have had a significant impact on scientific texts. Published articles were divided into three time periods based on the publication pattern and citations received: the first period (1953-1990), the second period (1991-2010), and the third period (2011-2020). There were few articles in the first period. Despite the small number of articles, the number of citations to them was significant. Another point in the review of citations during this period was the article published in 1971, which was well-cited by readers, which was about nurses' attitudes concerning elderly patients [29]. Data analysis over the last seven decades has shown that since the second period, the growth of articles has accelerated slightly and has grown significantly compared to previous years, perhaps because of the introduction of the Internet and online publication of articles after 1990. Moreover, with the fast development of mass media and technology, people receive overt medical data [30]. The rate of citation to articles in this period increased in proportion to the number of articles published. The peak of citations was in 1995. The growth rate of publications from the third period compared to the previous two periods rose significantly and had a rapid expansion. Antunez and Henry showed that the publication of geriatric nursing articles peaked in 2009 with sixty articles, and the articles received citations nearly 280 times [8]. One of the reasons that publications increased in this period could be that in 2009, several scholars and their colleagues created an alumni organization to use their academic and leadership training for advancing gerontological nursing [30]. The maximum growth of articles was in 2018. This indicates that in recent years, more researchers have been interested in researching this field and solving related problems and issues, and other potential reasons may be the increase in the aging world population and changes in countries' policies and their attention to issues related to the elderly and increasing medical care costs. It could relate to more researchers specializing in geriatric nursing. The spectrogram of the number of citations and papers has shown that although the volume of articles has increased slightly in the last decade, the citation rate has decreased. In other words, it has been less popular with other readers. In this period, the growth rate of articles shows a completely inverse relationship with the growth rate of citations. The quality of research in geriatric nursing should be considered.

The spectrogram of the number of citations and number of coauthors showed that, with an increase in the number of authors, the mean citations also increase. The size of research groups and the scientific impact in terms of citations considerably correlate with each other. Team size and citations have a close relationship, as confirmed by this study. This finding is supported by the results of another study that showed the median citations to articles increase with team size [31].

The current study indicated that the USA was a pioneer in this field. This was not unusual because the U.S. is the most active country in the study of geriatric nursing, and U.S. researchers rank first when it comes to scientific production in medicine, health, and nursing [30, 32, 33]. Additionally, it was revealed that the large scientific community, big research budget, higher national self-citation, desire to publish in national journals, and developed country are also contributing factors in the USA being a pioneer in this field [34, 35]. In the USA, the population age sixty-five years and over is estimated to nearly double over the next three decades, from 48 million to 88 million by 2050, and the need for more research in this field is felt. A decade ago, the Institute of Medicine published its groundbreaking report: Retooling for an Aging America: Building the Health Care Workforce (2008). This report emphasized the need to increase the number of health care providers competent in providing high-quality care for the ever-growing elderly population [36]. According to previous findings, it seems possible that these results are related to America's size and economic strength [27]. Based on the results, we can state that Brazil, Italy, England, France, Sweden, Netherlands, Germany, Australia, Canada, and the USA are among the countries that comprise 81% of all articles to geriatric nursing. Previous nursing literature shows that the term advanced practice is prevalent, and most of the current nursing literature comes from countries with a higher income (Australia, Canada, USA, Ireland, New Zealand, and South Africa) [37].

Moreover, countries such as the Netherlands, England, Canada, the USA, and Australia have a long history in nursing [9]. These factors could be why they are pioneers in geriatric nursing.

The results showed that Asian and African countries were not active in producing research in geriatric nursing. Since healthcare systems in these regions are rapidly evolving, it is essential to look at the scopes of practice of other nursing roles and how they interact with one another. Moreover, how these scopes evolve should be understood in lower-income countries in which regulatory processes are often underdeveloped [38]. In Asian countries, taking care of the elderly by their children at home is embedded in their culture. In such conditions, geriatric issues are less discussed at the societal level and, thus, are not paid that much attention to and are not considered a priority. Therefore, related research is scarce. In contrast to previous research [39], China was not among the pioneering countries, which supports this assumption. Seven out of ten institutes in the list of geriatric nursing research were from America. Based on the assessments of more than two decades in America, the results reveal the need for increasing the capacity of gerontological nursing in the USA on growing daily basis [30]. This is probably the reason why these institutes have tended towards geriatric nursing research. Secondly, the John A. Harford Foundation designed and implemented the Building Academic Geriatric Nursing Capacity Program in 2000 to emphasize the importance of gerontological nursing on society's health [40]. Moreover, the American Academy of Nursing plays a leading role in the development and implementation of geriatric nursing programs and creating the Building Academic Geriatric Nursing Capacity Program in American universities [40]. Many institutes and universities in America such as the Association for Gerontology in Higher Education, Gerontological Advanced Practice Nurses Association, National Hartford Center of Gerontological Nursing Excellence, the New York University's Rory Meyers, American Association for Colleges of Nursing, and the College of Nursing's Hartford Institute for Geriatric Nursing have strived to enhance geriatric nursing research. Shortly after, many American universities have made significant efforts to design educational frameworks to create stronger gerontological education for nurse generalists [41]. These initiatives have created many advantages for the elderly.

Canadian and Swedish institutions have played a prominent role alongside American institutions. Geriatric nursing is one of the fields in which scientific cooperation, especially at the international level, will help its development. The geographical distance seems to explain some of these international scientific collaborations. The closer the fellow researchers are to a geographical area, the more likely they are to form a research team. Twenty-eight papers were authored by Stig Karlsson, one of the most influential scientists in the field of geriatric nursing, from the Department of Nursing of Umea University. After Stig Karlsson, as the most prolific writer, the next ranks are held by writers from Belgium and Sweden. The results showed that half of the prolific authors were from the Scandinavian countries. The present study identified the willingness of Scandinavian writers, including Sweden, Belgium, and Finland, to study geriatric nursing. Perhaps the reason for this is the adoption of supportive policies at the top management levels in these countries. Top-level managers and politicians in Scandinavia have predicted that nursing competence at higher levels is necessary for meeting the complex needs of the elderly [42]. More and more Scandinavian researchers have begun to focus only on geriatric nursing studies. The support of the Scandinavian countries for geriatric nursing programs is evident in the scientific activities of their researchers. The researchers introduced in the present study are among the leaders in geriatric nursing research, and following their research can be both instructive for other interested researchers and can be used to design future research.

The results showed that the authorship clusters formed are usually influenced by the geographical distance and the joint institution. For example, as seen in Table 3, the top researchers are in the pale green cluster from Umea University in Sweden while the pale orange cluster from Katholieke Universiteit Leuven in Belgium. Authors who are geographically close to each other are more likely to form scientific research collaborations.

By studying the author keywords, it can be inferred that researchers are more motivated to study themes such as dementia, malnutrition, education, depression, and Alzheimer's disease. In all periods, geriatric nursing researchers have studied these topics more than any other themes. By extracting and introducing emerging topics, we can highlight future directions. Therefore, future researches with a focus on COVID-19, interprofessional, locomotive syndrome, public health, and emergency nursing are suggested. The most cited article was “A multidisciplinary intervention to prevent the readmission of elderly patients with congestive-heart-failure” in 1995 with 1571 citations. It showed that a nurse-directed, multidisciplinary intervention could increase the quality of life and reduce hospital use and medical costs for elderly patients with congestive heart failure. Most cited papers were published in the second period (1991-2010). In this period, the growth of articles and citations shows a fixed pattern. The topics of this period are still of interest to researchers and readers. The articles of this period show better performance than the first and third periods. In addition, it shows that the flow of geriatric nursing information has been higher during this period. Since citations increase over time, older articles have a longer time to gain citations and have more advantage over their young peers [43]. No rapid changes were observed in this field. The changes were more gradual.

The journals that publish the most geriatric nursing articles were identified. The present study showed that active journals all have a high impact factor based on the journal citation reports (JCR). Researchers can access a large part of geriatric nursing texts by following the journals listed. The American Geriatrics Society has a professional journal for geriatric nursing scientists. One journal in the field of psychology (International Psychogeriatrics) in the active journals list emphasizes the importance of psychology in geriatrics.

The presence of articles in different categoric quartiles may confirm the claim that the field of aging is multidisciplinary. It is also inferred from the distribution of articles in different quartiles that research with the Team Science approach in geriatric nursing will be very fruitful. Issues related to geriatrics will be solved using a multidisciplinary approach.

## 5. Strength and Limitations

The current study is the first bibliometric study to investigate research activity on geriatric nursing. Geriatric nursing articles were reviewed over a long period of nearly seven decades. The names of the authors of the articles may be written in different ways; to achieve the correct results, the authors who used different names were joined together and then analysed. We only used the WoS database; therefore, future studies can use the SCOPUS database or Google Scholar. Another limitation was that only English language research was included in the study.

## 6. Conclusions

In the current study, scientific literature on geriatric nursing was assessed and analyzed. Geriatric nursing is an established and a mature field and has received increasing attention in the last decade. It is necessary to pay more attention to the quality of texts in recent years, in line with the slight growth of geriatric nursing texts. The USA and American institutions play an active role in geriatric nursing. Aging populations have affected a wide range of countries in the world, and in addition to leading countries in this field, some developing countries have paid attention to geriatric nursing by publishing scientific articles in recent years. Geriatric nursing is one of the subject areas that can flourish through international collaborations and contributions of authors. By introducing research themes, highly cited articles, and active journals, the results can be useful for future research. Policymakers, especially in developing countries, should pay attention to geriatric nursing as a specialty of nursing both as a discipline of research and as a profession to solve aging issues they would face considering the increasing elderly population.

## Figures and Tables

**Table 1:** Top ten active countries in papers on Geriatric Nursing (1990–2020).

Country	Frequency	% *N* = 4923
USA	1862	37.822%
Canada	307	6.236%
Australia	292	5.931%
Germany	253	5.139%
Netherlands	251	5.099%
Sweden	245	4.977%
France	228	4.631%
England	202	4.103%
Italy	185	3.758%
Brazil	160	3.250%

**Table 2:** Top ten active institutions in publishing papers on Geriatric Nursing (1990–2020).

Institution	Country affiliation	Frequency	% *N* = 4923
New York University	USA	97	1.970%
University California Los Angeles	USA	93	1.889%
University Toronto	Canada	76	1.544%
Karolinska Institutet	Sweden	65	1.320%
The University of California, San Francisco	USA	65	1.320%
University Minnesota	USA	62	1.259%
University of Pennsylvania	USA	61	1.239%
University of Pittsburgh	USA	61	1.239%
Umeå University	Sweden	58	1.178%
The University of North Carolina	USA	55	1.117%

**Table 3:** Top ten active authors in papers on Geriatric Nursing (1990–2020).

Authors	Country affiliation	Frequency	% *N* = 4923
Karlsson S	Sweden	28	0.569%
Milisen K	Belgium	26	0.528%
Sandman PO	Sweden	26	0.528%
Bernabei R	USA	25	0.508%
Gustafson Y	Sweden	25	0.508%
Rubenstein LZ	France	25	0.508%
Mezey M	USA	24	0.488%
Vellas B	France	23	0.467%
Capezuti E	USA	22	0.447%
Pitkala KH	Finland	21	0.427%

**Table 4:** 10 most cited papers on Geriatric Nursing (1990–2020).

Title	Year	Citations
A multidisciplinary intervention to prevent the readmission of elderly patients with congestive-heart-failure	1995	1571
Rating chronic medical illness burden in geropsychiatric practice and research - application of the cumulative illness rating-scale	1992	1283
The mini nutritional assessment (MNA) and its use in grading the nutritional state of elderly patients	1999	874
Hazards of hospitalization of the elderly	1993	848
Hazards of hospitalization of the elderly	1996	822
Management of pain in elderly patients with cancer	1998	752
The effects of exercise training on elderly persons with cognitive impairment and dementia: A meta-analysis	2004	741
Diagnosis and treatment of Alzheimer disease and related disorders - Consensus statement of the American Association for Geriatric Psychiatry, the Alzheimer's Association, and the American Geriatrics Society	1997	696
Validation of the cumulative illness rating-scale in a geriatric residential population	1995	659
Functional Status of Elderly Adults before and after Initiation of Dialysis	2009	590

**Table 5:** 20 active journal papers on Geriatric Nursing (1990–2020).

Journal	Frequency	% *N* = 4923	Country	Impact factor	Quartile in category
Journal of the American Geriatrics Society	373	7.57	USA	4.180	Q1
Journal of the American Medical Directors Association	130	2.64	USA	4.367	Q1
Geriatric Nursing	106	2.15	USA	4.367	Q1
Journal of Clinical Nursing	93	1.88	England	1.972	Q1
European Geriatric Medicine	93	1.88	USA	1.232	Q4
International Journal of Geriatric Psychiatry	93	1.88	England	2.675	Q3
BMC Geriatrics	86	1.74	England	3.077	Q2
Journal of Gerontological Nursing	84	1.70	USA	1.016	Q3
Journal of Advanced Nursing	80	1.62	USA	2.561	Q1
Archives of Gerontology and Geriatrics	78	1.58	Ireland	2.128	Q3
Journal of Nutrition Health and Aging	68	1.38	France	2.791	Q3
Age and Ageing	64	1.30	England	4.902	Q1
Educational Gerontology	62	1.25	USA	0.621	Q4
International Journal of Nursing Studies	60	1.21	England	3.783	Q1
Revista da Escola de Enfermagem da USP	54	1.09	Brazil	0.798	Q4
International Psychogeriatrics	53	1.07	USA	2.940	Q1
Geriatrics and Gerontology International	49	0.99	Japan	2.022	Q3
Aging Clinical and Experimental Research	46	0.93	Italy	2.697	Q3
Aging and Mental Health	46	0.93	England	2.478	Q3
American Journal of Geriatric Psychiatry	45	0.91	USA	3.393	Q2

## Data Availability

The data used to support the findings of this study are included within the article.
